# ARAP3 Functions in Hematopoietic Stem Cells

**DOI:** 10.1371/journal.pone.0116107

**Published:** 2014-12-26

**Authors:** Yiwen Song, Jing Jiang, Sonja Vermeren, Wei Tong

**Affiliations:** 1 Department of Pediatrics, University of Pennsylvania Perelman School of Medicine, Philadelphia, Pennsylvania, United States of America; 2 Division of Hematology, Children's Hospital of Philadelphia, Philadelphia, Pennsylvania, United States of America; 3 MRC Centre for Inflammation Research, The Queen's Medical Research Institute, The University of Edinburgh, United Kingdom; French Blood Institute, France

## Abstract

ARAP3 is a GTPase-activating protein (GAP) that inactivates Arf6 and RhoA small GTPases. ARAP3 deficiency in mice causes a sprouting angiogenic defect resulting in embryonic lethality by E11. Mice with an ARAP3 R302,303A mutation (*Arap3^KI/KI^*) that prevents activation by phosphoinositide-3-kinase (PI3K) have a similar angiogenic phenotype, although some animals survive to adulthood. Here, we report that hematopoietic stem cells (HSCs) from rare adult *Arap3^KI/KI^* bone marrow are compromised in their ability to reconstitute recipient mice and to self-renew. To elucidate the potential cell-autonomous and non-cell-autonomous roles of ARAP3 in hematopoiesis, we conditionally deleted *Arap3* in hematopoietic cells and in several cell types within the HSC niche. Excision of *Arap3* in hematopoietic cells using *Vav1-Cre* does not alter the ability of ARAP3-deficient progenitor cells to proliferate and differentiate *in vitro* or ARAP3-deficient HSCs to provide multi-lineage reconstitution and to undergo self-renewal *in vivo*. Thus, our data suggest that ARAP3 does not play a cell-autonomous role in HSPCs. Deletion of *Arap3* in osteoblasts and mesenchymal stromal cells using *Prx1-Cre* resulted in no discernable phenotypes in hematopoietic development or HSC homeostasis in adult mice. In contrast, deletion of *Arap3* using vascular endothelial cadherin (*VEC* or *Cdh5*)-driven *Cre* resulted in embryonic lethality, however HSCs from surviving adult mice were largely normal. Reverse transplantations into *VEC*-driven *Arap3* conditional knockout mice revealed no discernable difference in HSC frequencies or function in comparison to control mice. Taken together, our investigation suggests that despite a critical role for ARAP3 in embryonic vascular development, its loss in endothelial cells minimally impacts HSCs in adult bone marrow.

## Introduction

Hematopoietic stem cells (HSCs) are the critical source of all blood cells. Their potential for self-renewal and multi-lineage repopulation sustains the rapid turnover of the blood system throughout life. The first HSC arises from the hemogenic endothelium in the Aorta-Gonad-Mesonephros (AGM) region of the embryo and subsequently colonizes the fetal liver [26]. In the adult mouse, HSCs reside in complex bone marrow (BM) niches that are not mutually exclusive. Extensive research has shown that HSC perivascular and osteoblastic niches are comprised of endothelial cells, mesenchymal stromal cells, osteoblasts, sympathetic nerves and non-myelinating Schwann cells [1]–[3].

HSC functions are tightly regulated by a plethora of extrinsic and intrinsic regulatory pathways. One such family of regulators is the Rho family of GTPases, molecular switches that cycle between an active guanosine triphosphate (GTP)-bound form and an inactive guanosine diphosphate (GDP)-bound form [4], [5]. Rho GTPases play pivotal roles in hematopoietic stem and progenitor cell (HSPC) actin cytoskeletal reorganization [6]–[13], with recent genetic studies expanding our knowledge of their roles to include HSC self-renewal, multi-lineage differentiation, homing/migration, proliferation, cytokinesis and survival [14]–[19]. GTPase-activating proteins (GAPs) stimulate the hydrolysis of bound GTP to GDP, thereby inactivating GTPases. GAPs, such as p190B RhoGAP, have been shown to be important regulators of HSC engraftment and interaction with its microenvironment [20], [21].

ARAP3 is a dual Arf and Rho GTPase-activating protein that was first identified in porcine leukocytes for its ability to bind to phosphatidylinositol (3,4,5)-triphosphate (PIP_3_) [22]. ARAP3 contains two distinct GAP domains that accelerate the rate of GTP hydrolysis to attenuate Arf6 and RhoA signaling [23], [24]. Previous *in vitro* studies found that either exogenous ARAP3 expression in epithelial cells or RNAi-mediated ARAP3 depletion in endothelial cells disrupts F-actin or lamellipodia formation, respectively, resulting in a cell rounding phenotype and failure to spread [25], [26]. This implies that ARAP3 controls Arf6 and RhoA in a tightly regulated fashion, and that maintaining precise regulation of ARAP3 activity is crucial to actin organization in the cell. RhoA has been characterized *in vivo* to regulate migration and chemotaxis of mature hematopoietic cells [27], [28], as well as HSPC engraftment, multi-lineage repopulation and cell survival [9], [14], [15], while the role of Arf6 in hematopoiesis is largely unknown.

In mice, ARAP3 is most highly expressed in the endothelium and bone marrow, and has been found to be critical to vascular development [29], [30]. Germline deletion or *Tie2-Cre*-mediated deletion of *Arap3* in mice leads to embryonic lethality by E11 due to defects in sprouting angiogenesis of the endothelium [29]. Since HSCs arise from the hemogenic endothelium during embryonic development around E10.5 [31], and give rise to all subsequent hematopoietic cells in the fetal liver and in the adult BM, this genetic model precludes further studies of ARAP3 function in definitive hematopoiesis and HSC function. Conditional *Arap3* deletion in neutrophils has been shown to alter their adhesion-dependent functions [32], [33], but the role of ARAP3 in HSPCs has yet to be defined.

ARAP3 is a phosphoinositide 3-OH kinase (PI3K)- and Rap- regulated GAP that is recruited to the plasma membrane in a PIP_3_-dependent fashion. PI3K-dependent activation of ARAP3 involves binding of its two most N-terminal pleckstrin homology (PH) domains to PIP_3,_ a lipid second messenger generated downstream of PI3K. This drives recruitment of ARAP3 to the plasma membrane to facilitate interaction with its GTPase substrates [34]. PIP_3_ binding is ablated when a tandem arginine to alanine mutation is introduced at residues R302,R303 in the first PH domain of ARAP3, preventing ARAP3 activation and recruitment to the plasma membrane [29]. *Arap3^R302,303A/R302,303A^* knock-in mutant mice (here referred to as *KI/KI*) phenocopy *Arap3* null mice, suggesting an essential role for PI3K-dependent activation of ARAP3 [29].

In this study, we first investigate ARAP3 function in adult hematopoiesis using *KI/KI* ^−^mice, since about 2% of *KI/KI* mice are viable [29], [33]. We report that *KI/KI* HSCs are compromised in their ability to repopulate and self-renew in serial transplantation assays. To elucidate potential cell-autonomous and non-cell-autonomous roles for ARAP3 in HSC function, we selectively delete *Arap3* in the hematopoietic compartment, and in endothelial and stromal cells of the HSC niche, respectively, using *Cre* driven by suitable promoters (*Vav1*, *Prx1*, *Cdh5*/*VE-cadherin*). Using these genetic models, we report that ARAP3 does not play a major role in regulating HSPC functions.

## Results

### Arap3 R302,303A mutation impairs HSC functions

**Figure 1:**
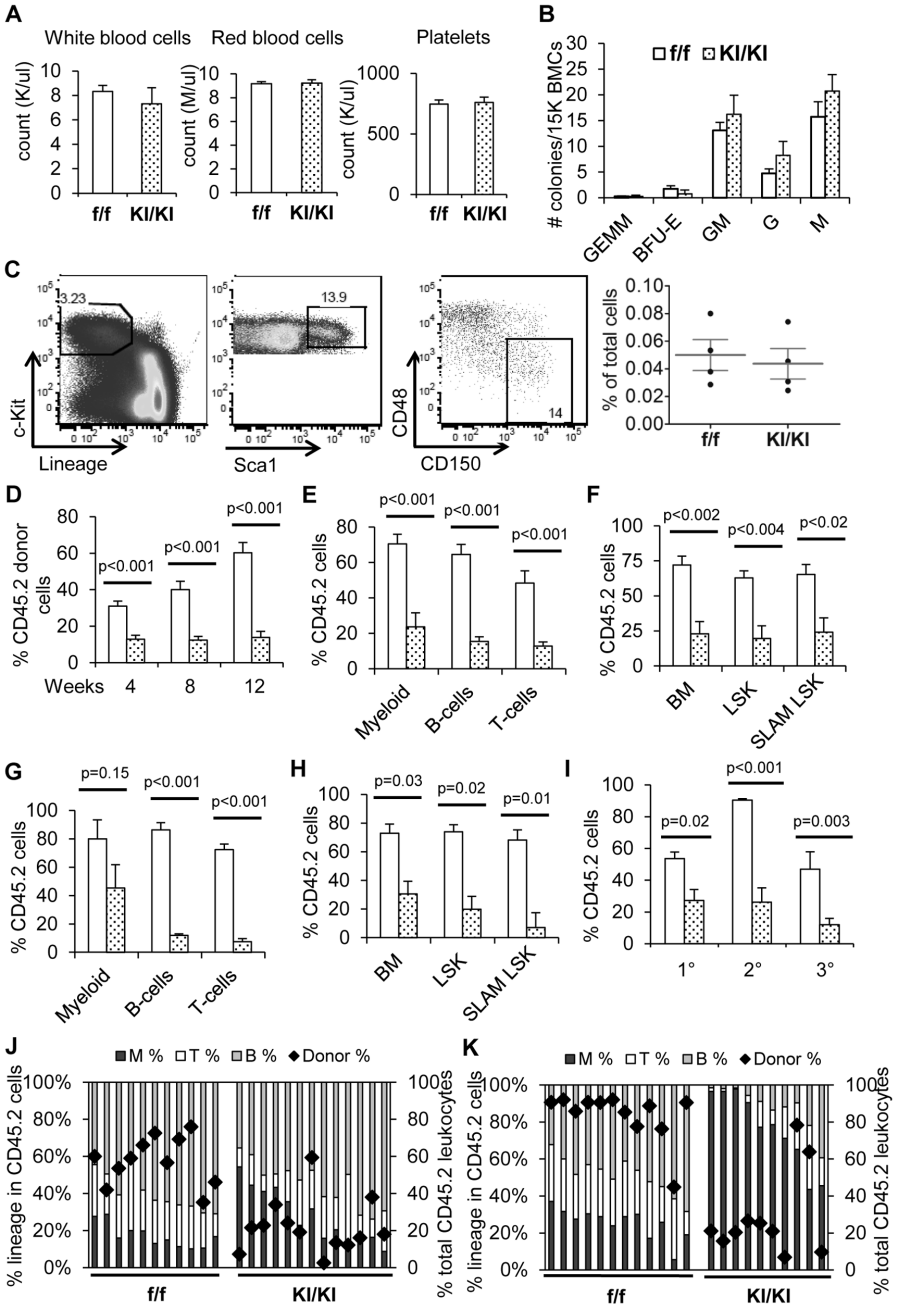
ARAP3 R302,303A mutation impairs HSC function. (A) Complete blood counts (CBC) of *f/f* control mice (white bars) and *KI/KI* mutant mice (dotted black bars). n = 4. (B) Colony-forming cell (CFC) assays of *f/f* and *KI/KI* bone marrow cells enumerated after 11 days in culture. Colonies identified as multipotent myeloid progenitors (GEMM), erythroid (BFU-E), granulocyte-monocyte progenitors (GM), granulocyte (G), or monocyte (M). n = 3. (C) The percentage of SLAM LSK population enriched for long-term hematopoietic stem cells in *f/f* and *KI/KI* bone marrow, gated on the Lin^−^c-Kit^+^Sca1^+^ (LSK) population, followed by SLAM markers CD48^−^CD150^+^. (D–K) Transplantation of LSK cells from *f/f* and *KI/KI* donor mice. Results were pooled from two separate experiments. (D) Peripheral blood in the recipients was assessed at 4, 8, and 12 weeks after primary transplant by flow cytometry for the percentage of donor-derived leukocytes. (E,H) Donor contribution to myeloid, B-cell, and T-cell compartments in the peripheral blood at the end of the primary (E) or secondary (H) transplant was analyzed by flow cytometry. (F,H) Donor percentages in the total BM, LSK, and SLAM LSK compartments (CD48^−^CD150^+^LSK) were enumerated by flow cytometry, 16 weeks post primary (F) or secondary (H) transplantation. (I) Peripheral blood reconstitution in serial transplants was assayed by flow cytometry. 1°: primary; 2°: secondary; 3°: tertiary BMT. (J,K) Bars show lineage distributions within donor-derived cells of individual recipient mice (left Y-axis), while diamond symbols indicate total donor leukocyte percentages (right Y-axis) in the peripheral blood at the end of the primary (J) or secondary (K) BM transplants. T: CD3^+^; B: CD19^+^; M: Mac1^+^. Graphs show mean ±SEM. P-values determined by two-tailed Student's t-test.

To study whether ARAP3 function affects adult hematopoiesis and HSC function, we studied *KI/KI* mice expressing mutant ARAP3 R302,303A. This point mutation interferes with the ability of ARAP3 to bind PIP_3_ and its subsequent activation by PI3K [29], [33]. Most *KI/KI* mutant mice die embryonically at E11 [29], but a small subset (∼2%) was viable and fertile when the expected birth ratio was 25% (Table 1). By 8–12 weeks of age, these mice were indistinguishable from their littermate controls in gross appearance as well as by phenotypic characterization of their peripheral blood (Fig. 1A). *KI/KI* BM also showed normal progenitor cell numbers as determined by colony-forming cell (CFC) assays (Fig. 1B), and normal HSC frequencies as determined by flow cytometry using SLAM family surface markers, CD48^−^CD150^+^LSK (Lin^−^Sca1^+^cKit^+^) [35] (Fig. 1C).

**Table 1:** *KI/KI* live birth rates.

	*KI/KI*	*KI/+*	+/+
*KI/+ x KI/+*	2/110 (1.8%)	68/110 (61.8%)	40/110 (36.4%)
Expected ratios	25%	50%	25%
*KI/+ x KI/KI*	6/36 (16.7%)	30/36 (83.3%)	–
Expected ratios	50%	50%	–

Breeding pair genotypes are displayed in the left column. Genotypes of expected pups are labeled at the top of each column. Birth rates of each genotype are displayed as a percentage and fraction of total pups born alive. Expected Mendelian ratios of each genotype are listed below each cross.

To study the function of these mutant HSPCs, purified LSK cells from *KI/KI* mutant mice or control mice were injected with competitor bone marrow cells into each irradiated recipient mouse. Reconstitution in individual recipient mice was followed every 4 weeks post-transplant. We found that *KI/KI* LSKs displayed a significantly lower donor chimerism in all lineages of the peripheral blood from recipient mice (Figs. 1D and 1E). Donor-derived cells in the BM, LSK, and SLAM LSK (CD48^−^CD150^+^LSK) compartments were significantly lower in mice transplanted with *KI/KI* cells, in comparison to control cells (Fig. 1F). Four months after the primary transplant, BM cells were harvested and 2×10^6^ total BM cells were injected into secondary irradiated recipient mice. Tertiary transplants were performed similarly. Peripheral blood and BM HSC reconstitution after each transplant was analyzed by flow cytometry. We found that the defects in reconstitution of *KI/KI* cells were exacerbated upon serial transplantations, indicating compromised HSC self-renewal (Figs. 1G–1I). Interestingly, a myeloid bias in the multi-lineage reconstitution of serially transplanted recipients arose within *KI/KI* reconstituted mice (Figs. 1J and 1K), reminiscent of aged HSCs [36]–[38].

### ARAP3 is dispensable for steady-state hematopoiesis

Our data from the knock-in mice prompted us to further elucidate the role of ARAP3 in regulating HSC function using ARAP3 conditional knockout (CKO) mice. To study if ARAP3 plays a cell-autonomous role in hematopoietic cells, we crossed *Arap3^flox/flox^* (*f/f*) mice with mice expressing a *Vav1* promoter-driven *Cre*. *Vav1* expression begins around E11.5, and is fully turned on and expressed in greater than 99% of hematopoietic cells by E13.5, thereby excising floxed alleles in most, if not all, fetal liver and adult hematopoietic cells [39].

**Figure 2:**
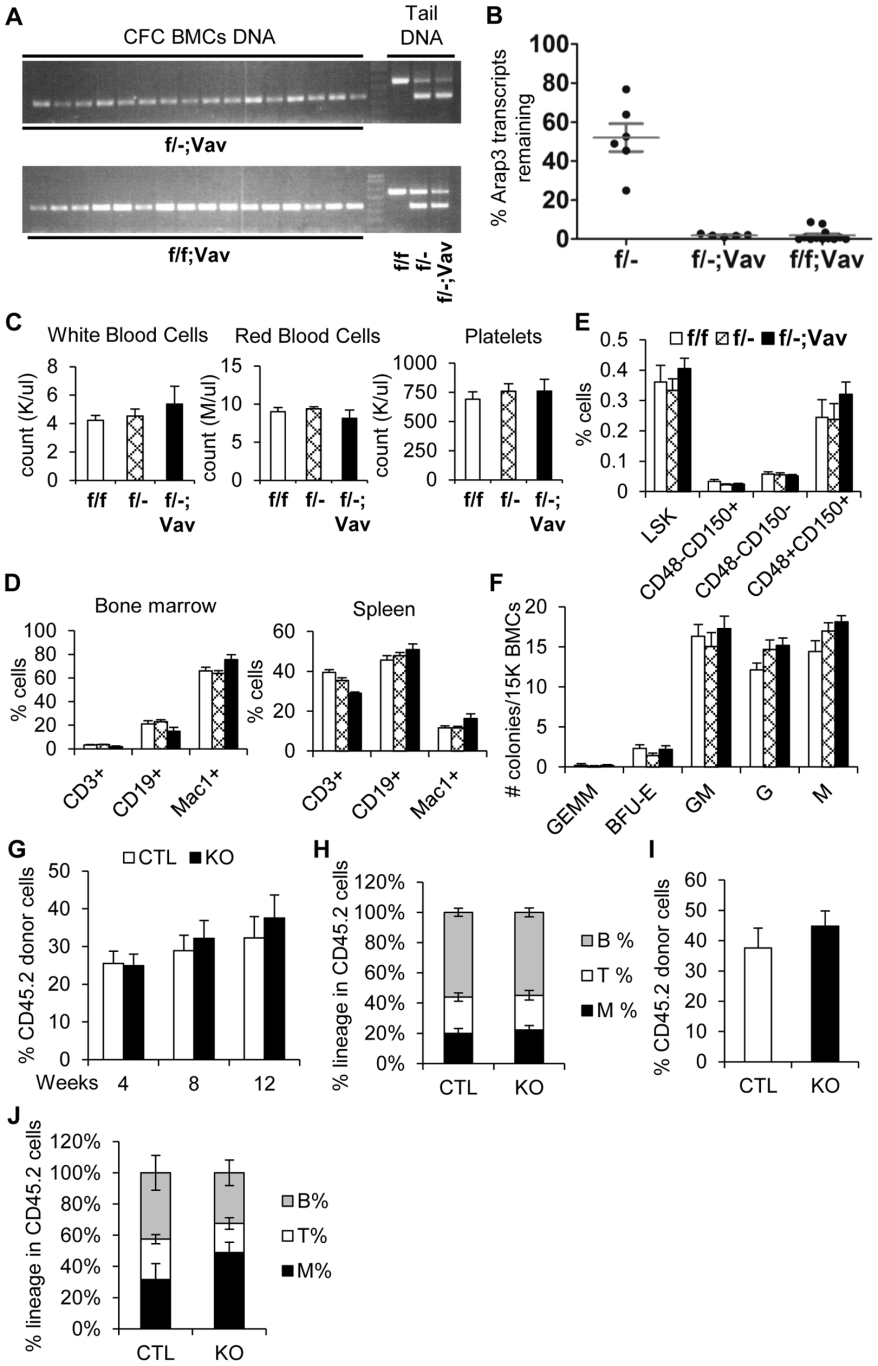
Hematopoietic-specific deletion of Arap3 using Vav1-Cre does not affect HSC repopulation or self-renewal. (A) Representative PCR genotyping results of individual colonies from CFC assays of *f/−;Vav* and *f/f;Vav* CKO mice. Controls are tail DNA isolated from *f/f, f/−* and *f/−;Vav* mice, showing the *Arap3* floxed band (top band) and the *Arap3* deleted band (bottom band). (B) *Arap3* RNA transcript levels from BM of *f/−*, *f/f;Vav*, and *f/−;Vav* mice were assayed by qRT-PCR and first normalized to *Gapdh* levels. The graph shows the relative *Arap3* transcripts remaining in the CKO mice compared to that in *f/f* controls. Each symbol represents an individual mouse; horizontal lines indicate mean ±SEM levels. (C) CBC of *f/f* (white bars), *f/−* (crosshatch bars), and *f/−;Vav* (black bars) mice. n = 8. (D) Percentage of various cell populations in the BM and spleen of *f/f*, *f/−*, and *f/−;Vav* mice was analyzed by flow cytometry, as defined by the surface markers indicated. n = 5. (E) Percentage of various HSPC populations in the BM from *f/f*, *f/−*, and *f/−;Vav* mice was quantified using flow cytometry, defined by Lin^−^Sca1^+^c-Kit^+^ (LSK) and SLAM markers CD48 and CD150. n = 5. (F) CFC assays of *f/f*, *f/−*, and *f/−;Vav* BM cells were enumerated after 11 days in culture. n = 8. (G–J) Primary and secondary transplantation of LSK cells from control (CTL  =  *f/f* and *f/−*) and knockout (KO  =  *f/f;Vav* and *f/−;Vav*) donor mice. Data pooled from 3 independent experiments. n = 8–15. (G) Peripheral blood in the recipients was assessed at 4, 8, and 12 weeks after primary transplant by flow cytometry for the percentage of donor-derived cells. White bars represent control donors and black bars represent CKO donors. (I) Peripheral blood of recipients assessed at the end of the secondary transplant for donor chimerism by flow cytometry. (H, J) Bars show lineage distributions within donor-derived cells of individual recipient mice at the end of the primary (H) and secondary (J) transplants. T: CD3^+^; B: CD19^+^; M: Mac1^+^. Graphs show mean ±SEM. P-values determined by Student's t-test.

To measure the deletion efficiency at the DNA level, we cultured 1.5×10^4^ unfractionated BM cells from either *Arap3^flox/flox^;Vav1-Cre^tg^* (*f/f;Vav*) CKO or *f/f* control mice in semi-solid methylcellulose cultures. Each progenitor cell gives rise to an individual colony, from which DNA was isolated to measure *Arap3* deletion efficiency on a clonal basis. Clonal analysis by polymerase-chain reaction (PCR) genotyping showed near 100% *Vav1-Cre*-mediated excision of *Arap3* (S1A Fig.). We also measured *Arap3* deletion efficiency at the transcript level using quantitative real-time PCR (qRT-PCR). *f/f;Vav* showed a greater than 95% deletion of *Arap3* transcripts in the BM when compared to *f/f* control mice (S1C Fig.). In contrast, the transcript levels of the other ARAP family members, *Arap1* and *Arap2*, remained unchanged in the BM (S1D Fig.). To ensure a more complete deletion of ARAP3 in hematopoietic cells, we generated *Arap3^flox/−^;Vav1-Cre^tg^* (*f/−;Vav*) mice. These mice showed 100% deletion in all *f/−;Vav* mice we examined at both DNA and RNA levels (Figs. 2A and 2B). We found that both *f/f;Vav* and *f/−;Vav* mice were born at normal Mendelian ratios and appeared grossly normal (data not shown). Adult *f/f;Vav* and *f/−;Vav* mice showed normal peripheral blood composition (S2A and 2C Figs.). The lineage distribution of hematopoietic tissues as analyzed by flow cytometry in *Arap3* CKO mice was comparable to that of their littermate controls (S2B and 2D Figs.). Our data suggest that ARAP3 is dispensable for steady-state hematopoiesis in the adult mouse.

### ARAP3 does not play a cell-autonomous role in HSCs

We next examined if ARAP3 affects primitive hematopoietic compartments. We found that *f/−;Vav* mice had a normal distribution of phenotypic HSPCs within the LSK compartment, as determined by SLAM markers (Fig. 2E). Furthermore, *Arap3* deficiency in BM cells did not affect hematopoietic progenitor cell proliferation or differentiation in CFC assays (Fig. 2F and S2C Fig.). Of note, when purified *f/f;Vav* LSKs were plated, they exhibited abilities to form various types of colonies with normal frequencies (S2D Fig.) and morphology (not shown). In contrast, *Arap3^−/−^* neutrophils from the CKO mice exhibited enhanced polyRGD-induced adhesion (S2E Fig.), as previously published [32]. These data together suggest the cell-intrinsic functions of ARAP3 are limited to more differentiated myeloid cells, rather than in the immature HSPC populations.

We next assessed whether ARAP3 plays a role in HSC function *in vivo* using competitive bone marrow transplantation (BMT) assays. LSK cells sorted from *f/f;Vav* mice or *f/f* littermate controls were transplanted into lethally-irradiated recipient mice and peripheral blood reconstitution was evaluated. There were no significant differences in multi-lineage reconstitution (S2H Fig.) or donor chimerism, either in the total blood cell population (S2F Fig.) or in myeloid/T-cell/B-cell lineages after primary BMT (S2G Fig.). Secondary transplants also showed normal multi-lineage reconstitution (S2I–S2K Figs.). Furthermore, serial transplantation of *f/−;Vav* LSKs showed no discernable differences in donor contribution (Figs. 2G and 2I) or multi-lineage reconstitution (Figs. 2H and 2J) when compared to littermate *Arap3^flox/−^* (*f/−*) or *f/f* control donors. Together, our data firmly established that ARAP3 does not cell-autonomously impact HSC homeostasis or function.

### ARAP3 is dispensable in Prx1-expressing stromal and osteoblastic cells for supporting steady-state hematopoiesis

**Figure 3:**
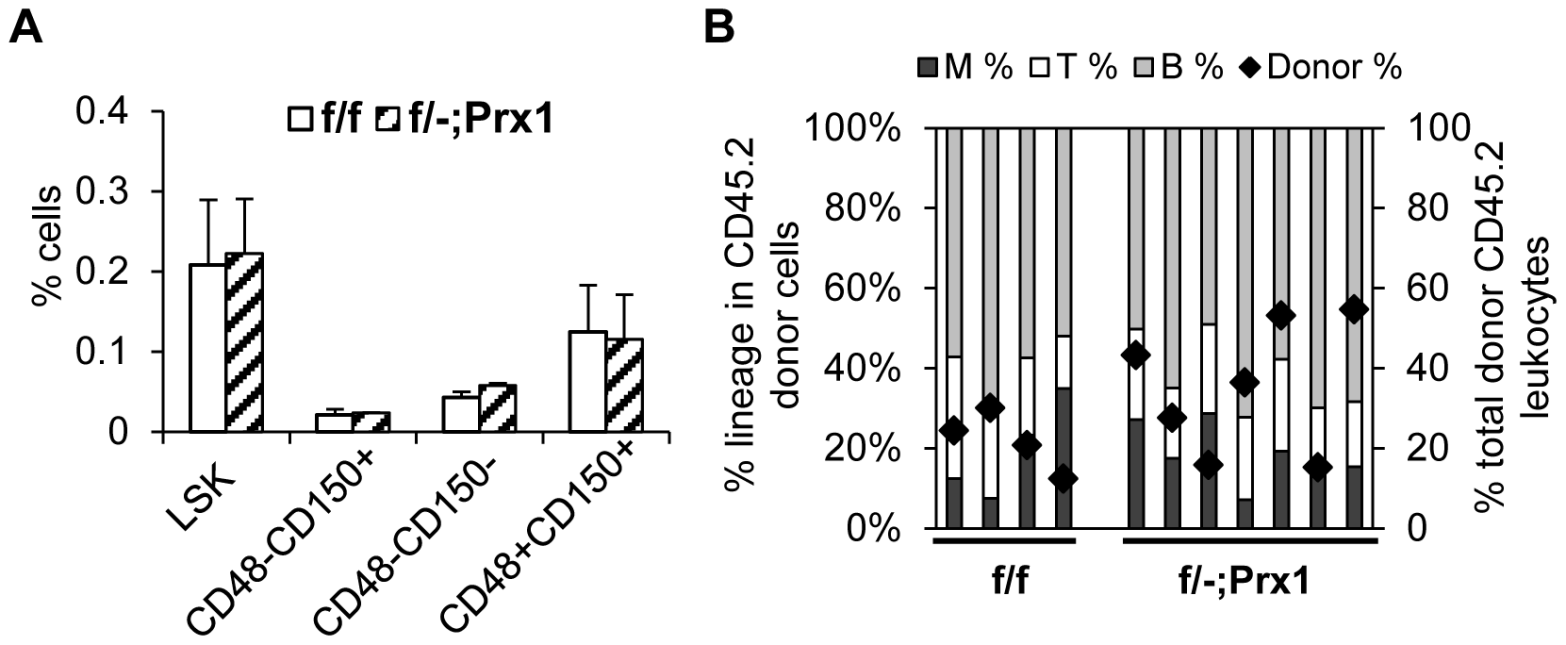
ARAP3 is dispensable in Prx1-expressing bone marrow niche cells for steady-state hematopoiesis. (A) BM cells from *f/f* control and *f/−;Prx1* CKO mice were quantified for the percentage of cells in various HSPC populations. Bars show mean ±SEM. n = 3. (B) LSK cells from *f/f* and *f/−;Prx1* donor mice were transplanted into irradiated recipient mice. Peripheral blood of individually reconstituted mice was assessed by flow cytometry at 12 weeks post-transplant for the distribution of cell lineages within donor-derived cells (bars, left Y-axis) and the percentage of total donor leukocytes (black diamonds, right Y-axis). P-values determined by Student's t-test.

We next investigated whether ARAP3 plays a role in the HSC niche, acting non-cell-autonomously to regulate HSCs. To study this, we deleted *Arap3* using *Prx1-Cre*, shown to induce excision in nearly all osteoblasts and 95% of perivascular stromal cells but not in endothelial cells [2], [40]. *Arap3^flox/−^;Prx1-Cre^tg^* (*f/−;Prx1*) mice were born alive in expected Mendelian ratios. Hematopoietic development of *f/−;Prx1* mice appeared normal as determined by CBC and lineage distribution in hematopoietic tissues, as well as by progenitor numbers and function in CFC assays (data not shown). *f/−;Prx1* mice also exhibited normal frequencies of phenotypic HSCs and progenitors as characterized by flow cytometric analysis (Fig. 3A). Using competitive BMT assays, we found that *f/−;Prx1* LSKs repopulated comparably to control *f/−* LSK cells (Fig. 3B). These data indicate that ARAP3 in *Prx1*-expressing niche cells is not a significant regulator of steady-state hematopoiesis or HSC homeostasis.

### ARAP3 expression in endothelial cells is important for embryonic development but not adult HSC functions

*Arap3* deletion in endothelial cells using *Tie2-Cre* resulted in embryonic lethality due to a cell-autonomous angiogenesis defect, though only with more robust excision on the *f/−*, but not *f/f*, background [29]. Since *Tie2-Cre* also shows some expression in stromal cells [41], [42], we utilized the vascular endothelial cadherin (*VEC* or *Cdh5*) promoter-driven Cre (*VEC-Cre*) that is found to be more endothelial cell-specific than *Tie2-Cre* [43], [44]. *Arap3^flox/flox^;VEC-Cre^tg^* (*f/f;VEC*) mice were born at expected Mendelian ratios (Table 2).

**Table 2:** *f/f;VEC* and *f/−;VEC* live birth rates.

	*f/f;VEC*	*f/+;VEC*	*f/f*	*f/+*
*f/f x f/+;VEC*	30/142 (21.1%)	37/142 (26.1%)	36/142 (25.3%)	39/142 (27.5%)
Expected ratios	25%	25%	25%	25%

	*f/−;VEC*	*f/f;VEC*	*f/−*	*f/f*
*f/− x f/f;VEC*	14/136 (10.3%)	30/136 (22.1%)	45/136 (33.1%)	47/136 (34.6%)
Expected ratios	25%	25%	25%	25%

Breeding pairs are labeled in the left column. Genotypes of expected pups are labeled at the top of each column. Birth rates are displayed as a percentage and fraction of total pups born alive. Expected Mendelian ratios are listed below each cross breeding.

**Figure 4:**
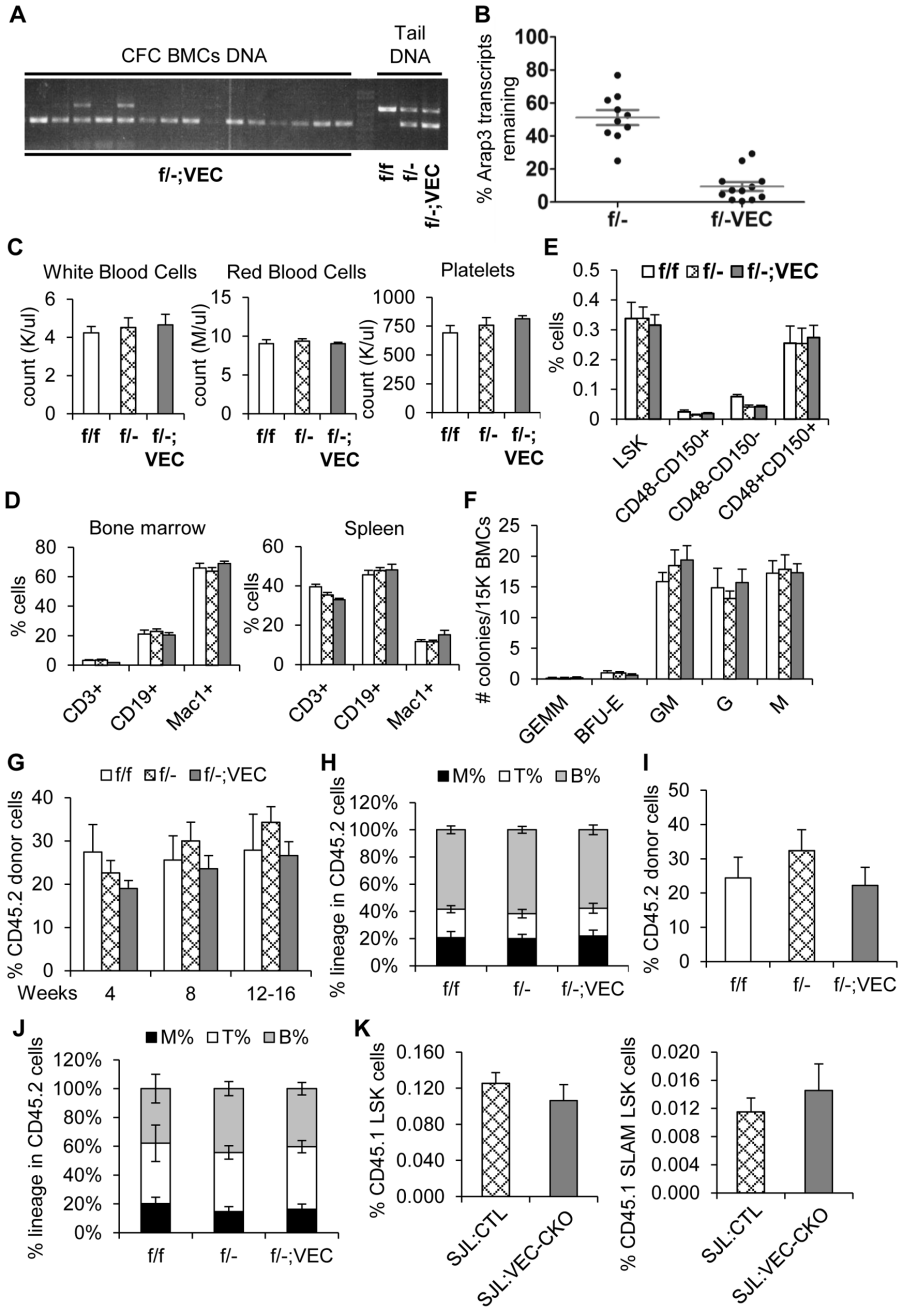
Arap3^flox/−^;VEC-Cre mice show largely normal HSC homeostasis and function. (A) Representative PCR genotyping results of individual colonies from CFC assays of *f/−;VEC* CKO BM cells are shown. Controls are tail DNA isolated from *f/f* and *f/−* control mice as well as *f/−;VEC* CKO mice, showing the *Arap3* floxed band (larger band) and the *Arap3* deleted band (smaller band). (B) *Arap3* RNA transcript levels from BM of *f/−* and *f/−;VEC* mice were assayed by qRT-PCR. The graph shows the relative *Arap3* transcript level to that in *f/f* controls. Each symbol represents an individual mouse; horizontal lines indicate mean ±SEM levels. (C) CBC of *f/f* (white bars) and *f/−* (crosshatch bars) control mice and *f/−;VEC* (gray bars) CKO mice. n = 5. (D) Percentage of various cell populations in the BM and spleen of *f/f*, *f/−*, and *f/−;VEC* mice was analyzed by flow cytometry. n = 5. (E) Quantification of the percentage of various HSPC populations in the BM from *f/f*, *f/−*, and *f/−;VEC* mice using flow cytometry. n = 5. (F) CFC assays of *f/f*, *f/−*, and *f/−;VEC* BM cells were enumerated after 11 days in culture. n = 5. (G–J) Serial BM transplantation of LSK cells from *f/f*, *f/−*, and *f/−;VEC* donor mice. Data pooled from 4 independent experiments. n = 18–24. (G,I) Peripheral blood in the recipients was assessed every 4 weeks post-primary transplant (G) and at the end of the secondary (I) transplant for the percentage of donor-derived cells. (H,J) Multi-lineage reconstitution was analyzed by flow cytometry. Bars show lineage distributions within donor-derived cells of individual recipient mice at the end of the primary (H) and secondary (J) transplantations. (K) **ARAP3 expression in BM endothelial cells is not required to support HSC homeostasis.** Reverse transplantation of wild-type CD45.1^+^ bone marrow cells into *f/f* and *f/−* control (SJL:CTL) or *f/f;VEC* and *f/−;VEC* CKO (SJL:VEC-CKO) mice were performed. Percentage of CD45.1^+^ LSK and SLAM LSK cells in the recipient bone marrow was quantified by flow cytometry 8 weeks post-transplantation. n = 9–15 pooled from two independent experiments. Graphs show mean ±SEM. P-values determined by Student's t-test.

To obtain a more complete deletion of *Arap3* in endothelial cells, we generated *Arap3^flox/−^;VEC-Cre^tg^* (*f/−;VEC*) mice. These CKO mice were born at a significantly reduced ratio (Table 2). The surviving CKO mice appeared grossly normal, and showed an approximate 88% excision efficiency (Fig. 4A), ranging from 80% to 96%, compared to an average 80% excision rate in *f/f;VEC* mice (S1B Fig.). By qRT-PCR, an approximate 9% *Arap3* transcripts remained in *f/−;VEC* BM (Fig. 4B), in contrast to 15% in *f/f;VEC* BM (S1C Fig.). In agreement with previously published data, this indicates that ARAP3 function in endothelial cells is essential for embryonic development.

Both *f/f;VEC* and surviving *f/−;VEC* adult mice had similar peripheral blood composition to their control littermates (S3A Fig. and Fig. 4C) and normal lineage distribution in hematopoietic tissues (S3B Fig. and Fig. 4D). These mice also exhibited a normal distribution of immature HSPC populations, with expected numbers of CD48^−^CD150^+^LSK cells (Fig. 4E). Hematopoietic progenitor numbers were normal as determined by CFC assays (Fig. 4F and S3C Fig.). We next examined HSC functions *in vivo* using competitive BMT assays. *f/f;VEC* LSKs showed comparable multi-lineage reconstitution to that of *f/f* controls in the primary (S3D–S3F Figs.) and secondary BMTs (S3G–S3I Figs.). Similarly, *f/−;VEC* LSK cells showed largely normal long-term reconstitution upon primary (Figs. 4G–4H) and secondary transplantation (Figs. 4I–4J) compared to *f/−* LSK cells.

To assess whether ARAP3 expression in BM endothelial cells is important to support HSC functions, we performed reverse transplantation of wild-type bone marrow cells into *f/f* and *f/−* control (SJL:CTL) or *f/f;VEC* and *f/−;VEC* CKO (SJL:VEC-CKO) recipient mice. HSC homeostasis or engraftment was not altered, and multi-lineage reconstitution of the hematopoietic compartment was unaffected by *Arap3* deletion in the host endothelial HSC niche (Fig. 4K and data not shown). Taken together, our investigation suggests that in spite of a critical role for ARAP3 during embryonic vascular development [29], [30], loss of ARAP3 in endothelial cells minimally impacts HSCs in the adult BM.

## Discussion

In the present study, we utilize *KI/KI* mice and generate three *Arap3* CKO mouse models to study ARAP3 functions in hematopoiesis of the adult mouse. We show that *KI/KI* mice exhibit defective HSC functions upon transplantation, indicating that PI3K-mediated ARAP3 function is important for HSCs. To elucidate the potential cell-autonomous and non-cell-autonomous roles of ARAP3 in HSCs, we conditionally deleted *Arap3* in hematopoietic cells and in several cell types in the HSC niche. *Arap3* null HSCs from *f/−;Vav* mice were functionally competent to repopulate the HSC pool and self-renew upon serial transplantation, revealing ARAP3 is not required in regulating HSC homeostasis or function in a cell-autonomous manner. Furthermore, ablation of ARAP3 in perivascular stromal cells, osteoblastic cells, and endothelial cells of the HSC niche did not alter HSC function or maintenance in *f/−;Prx1* and *f/−;VEC* mice. Reverse transplantation experiments strongly suggest that ARAP3 expression in the BM endothelial niche is not required to support HSC functions; however, future investigation is needed to assess the potential role of ARAP3 in BM mesenchymal and osteoblastic niches. Our study demonstrates that *f/−;VEC* mice displayed partial embryonic lethality, indicating a critical role for ARAP3 in endothelial cells during embryonic development but not in the adult BM niche for HSCs.

ARAP3 was first purified from porcine leukocytes for its PIP_3_ binding ability and is highly expressed in hematopoietic tissues, however our studies show that ARAP3 is not a critical cell-intrinsic regulator of hematopoiesis. ARAP3 does not play a cell-autonomous role in regulating HSC homeostasis or function. However, our observation does not rule out a cell-autonomous role for ARAP3 under stress conditions. This phenomenon has been seen in genetic studies of other proteins, such as SIRT1 [45], [46]. It is possible that a physiological challenge to the mice is necessary to elicit a specific function for ARAP3 in hematopoiesis. In our model, *Arap3* is excised in hematopoietic cells during early development using *Vav1-Cre*, and compensatory mechanisms may account for normal hematopoiesis in adult mice. It would be interesting to study whether acute loss of ARAP3 in hematopoietic cells of the adult mouse, such as with an inducible *Cre* system [47], would reveal a role for ARAP3 in hematopoiesis. Additionally, ARAP3 is part of a dual-GAP family that also includes ARAP1 and ARAP2. Although each family member targets different G-protein substrates and has different active functional domains [48]–[50], these three proteins may have overlapping and redundant roles in hematopoiesis, and may work in conjunction to regulate HSPC function. Thus, genetic studies of mice deficient in *Arap1* or *Arap2*, or with combination deletions of multiple ARAP proteins would clarify whether there is a significant role for the ARAP family of proteins in hematopoiesis.

We find that deletion of *Arap3* in stromal and osteoblastic cells using *Prx1-Cre* does not affect steady-state hematopoiesis or HSC homeostasis. Furthermore, deletion of *Arap3* in endothelial cells using *VEC-Cre* does not impact its ability to support wild-type HSCs. However, high efficiency of *Arap3* deletion in *f/−;VEC* mice results in embryonic lethality, suggesting ARAP3 plays an important role in developing endothelial cells, in agreement with previously published data [29], [30]. Since ARAP3 deficiency in embryonic endothelial cells disrupts angiogenic sprouting and vascular structure, this developmental-related dysregulation may indirectly affect HSC emergence, development, and maintenance in the adult mouse. However, our studies do not exclude the possibility that compensatory mechanisms may be upregulated in surviving *f/−;VEC* mice sometime between HSC emergence and adulthood. One approach to answer this question would be to induce the excision of *Arap3* in endothelial cells when ARAP3 is no longer required for vasculature development. Future investigations are warranted to examine the emergence of definitive hematopoietic progenitors and HSCs from the endothelium in the AGM region of *Arap3* null embryos and further our understanding of the role of ARAP3 in endothelial cells.

Our results demonstrate that the ARAP3 R302,303A mutation (*KI/KI*) that disrupts ARAP3 recruitment to the plasma membrane or activation by PI3K markedly impairs HSC function, while loss of ARAP3 does not. This could be due to compensatory mechanisms or changes in gene expression and signaling pathways of rare surviving *KI/KI* mice. Another possibility for compromised HSCs in *KI/KI* mice might be that the ARAP3 R302,303A mutant acts in a dominant negative manner to prevent translocation of other interacting players [51]–[55] to the plasma membrane, which may affect HSC function. For example, ARAP3 can bind the phosphatase SHIP2, a negative regulator of PI3K signaling [53], [54], [56], as well as CIN85 and Odin, both shown to be involved in receptor endocytosis and motility [51], [55], [57]–[59]. Although ARAP3 has not been shown to act as a scaffold for the plasma membrane translocation of any interacting proteins, and the *in vivo* relevance of these interactions remains to be tested, the fact that we see a cell-intrinsic defect with *KI/KI* HSCs but not with *f/−;Vav* HSCs in the BMT assays speaks in favor of this possibility. Lastly, it is possible that ARAP3 plays an important role in a cell type other than hematopoietic, endothelial, stromal, or osteoblastic cells to impact HSC function. One way of determining whether the phenotype of *KI/KI* HSCs is due to a cell-autonomous effect of the R302,303A mutant in HSCs would be to generate a *Vav1-Cre*-driven conditional *KI/KI* mouse to genetically study this question.

RhoA has been characterized *in vivo* to regulate migration and chemotaxis of mature hematopoietic cells [27], [28], as well as HSPC engraftment, multi-lineage repopulation and cell survival [9], [14], [15]. ARAP3 is not the only GTPase-activating protein that targets RhoA, and our data suggest it is not a major regulator of RhoA activation in HSPCs. Other GAPs, such as p190B RhoGAP, play important roles in mediating HSPC function through its inactivating activity on RhoA [20], [21]. Due to the large number of regulators for RhoA, it is likely that each acts in its own individual temporal- and spatial-specific manner. The possibility of redundancies between the various GAPs also exists [60], such that changing the dynamic by ablating one GAP is not enough to alter the process of normal hematopoiesis. It would be interesting to investigate whether deletion of multiple GAPs in hematopoietic cells would result in greater deficiencies than migration or engraftment alone.

As a dual GTPase-activating protein, ARAP3 targets Arf6 as well as RhoA. Arf6 has mostly been studied in non-hematopoietic cells with regard to its role in membrane trafficking and the cell actin cytoskeleton [61]–[63]. Like RhoA, it is actively involved in cell migration, adhesion, proliferation and cytokinesis [64]–[66]. However, the potential role of Arf6 in HSPCs and hematopoiesis has not been well established. One study showed that the decrease of active Arf6-GTP in platelets is critical to the activation of Rho GTPases that is necessary for cytoskeletal rearrangements preceding full platelet function [67]. It is important to further investigate and understand the role of Arf6 in hematopoiesis, particularly in HSCs.

ARAP3 has been implicated in the regulation and progression of several human diseases, including defense against bacterial infection, diabetes and gastric carcinoma, by capitalizing on the ability of ARAP3 to manipulate vesicle internalization and cell invasion [68]–[70]. Dysregulation of Rho family GTPases and their regulators have also been correlated with human blood disorders and tumorigenesis [71]–[75]. While aberrant expression of ARAP3 has not yet been found in blood disorders, its ability to regulate the actin cytoskeleton makes it a potential target for the dysregulation of homeostatic cell functions. Thus, continued study of ARAP3 in normal and abnormal hematopoiesis will be important to elucidate a more comprehensive understanding of its role in the blood system.

## Methods

### Generation of *Arap3* transgenic mice

*Arap3^flox/flox^* and *Arap3^KI/KI^* mice were generated as described previously [29]. *Vav1-Cre* mice were originally generated by Dr. Thomas Graf [39] and backcrossed to C57Bl/6J background for 8 generations. *VEC-Cre* mice were kindly provided by Dr. Nancy Speck [44] and backcrossed to C57Bl/6J background for 8 generations. These two strains of mice were crossed to generate *Arap3^flox/+^;Cre^tg^* mice, which were then crossed to *Arap3^flox/flox^* mice to generate *Arap3^flox/flox^;Cre^tg^* conditional knockout mice. Initial studies presented in S1–S3 Figs of *f/f;Vav* and *f/f;VEC* mice were done in mixed Bl6/129 background, while studies in main figures were later performed on a pure C57Bl/6J background following backcrossing for 8 generations. *Arap3^flox/+^* mice on the pure C57Bl/6J background were crossed with *CMV-Cre* mice on the C57Bl/6J background (The Jackson Laboratory) to generate *Arap3^+/−^* mice. These mice were used to generate *Arap3^flox/−^;Vav1-Cre^tg^* mice, *Arap3^flox/−^;VEC-Cre^tg^* and *Arap3^flox/−^;Prx1-Cre^tg^* conditional knockout mice (all on a pure Bl6 background) that will ensure a more complete deletion efficiency. *Prx1-Cre* mice on a C57Bl/6J background were purchased from The Jackson Laboratory.

The animal studies were carried out in strict accordance with the recommendations in the Guide for the Care and Use of Laboratory Animals of the National Institutes of Health. The protocol was approved by the Institutional Animal Care and Use Committee of the Children's Hospital of Philadelphia.

### PCR genotyping and qRT-PCR

For genotyping and clonal PCR analysis, primers were generated to detect wildtype, floxed and deleted alleles of *Arap3* using DNA isolated from CFU-C assays or mouse tissues. PCR reactions were performed on a BioRad thermal cycler using the following primers for *Arap3*: 5′-AGAGGCTCAGGACTAGAAGGACTA-3′ (Arap3_461F) and 5′-GGGCTGAGTAGAGACT GACGCGCC-3′ (Arap3_EcorV_F) and 5′-GAGGCCAGCCTGAGATAGATGAAACCC-3′ (Arap3_EcorV_R).

For quantitative real-time PCR, total RNA was isolated from FACS-sorted bone marrow cells, CFC assays or hematopoietic tissues using Trizol Reagent (Invitrogen Life Technologies) followed by isolation with the RNeasy Mini kit (Qiagen). cDNAs were produced using BioRad iScript kit and qRT-PCR reactions were performed on an Applied Biosystems 7900HT real-time PCR system using Sybr-Green detection with the following primers: *Arap3*: 5′–CCCTCTGACTGCCATCGA–3′ and 5′–ATTCCAGGTCATTACCGGCC–3′; *Arap1*: 5′-GATGCCGCACTGTCTGTAGCT-3′ and 5′-CTGCTCAAAGAGTGCCGTGTAC-3′; *Arap2*: 5′-CGGGACGAATGGCGTATTAG-3′ and 5′-TCTCGCCCTGAAACTGAAAGA-3′; *Gapdh*: 5′–GGAGCGAGACCCCACTAACA–3′ and 5′–TTCACACCCATCACAAACAT–3′. The transcript levels in CKO mice were first normalized to *Gapdh* levels, then expressed as a percentage of normalized levels in control *f/f* mice.

### Complete blood counts

Peripheral blood was collected by retro-orbital bleeding into capillary blood collection tubes with EDTA (BD). CBC analysis was performed using the mouse setting on a HemaVet 950 machine (Drew Scientific).

### Cell sorting and flow cytometry

Cells from either peripheral blood or hematopoietic tissues were lysed of red blood cells, then stained with surface markers on ice and washed in PBS with 2% bovine calf serum. Surface markers used to identify cell populations are: CD3e-PE for T-cells, CD19-APC for B-cells, Gr1-PE and Mac1-APC for myeloid cells (eBiosciences), and propidium iodide for viability. This method was also used for peripheral blood analysis of transplanted mice with the addition of CD45.1-PE-Cy7 and CD45.2-FITC antibodies. Flow cytometry was performed on a FACS Canto analyzer (BD).

For HSPC analysis, total bone marrow cells were flushed from femurs, tibias and iliac crests of mice. Single-cell suspensions were lysed of red blood cells and stained with the following primary antibodies: biotinylated lineage cocktail (B220, CD4, CD5, CD8, CD19, IL-7R, Gr1, Mac1, Ter119), Sca1-PerCP-Cy5.5, cKit-APC-Cy7, CD48-FITC, and CD150-PE-Cy7 (eBiosciences). Cells were then washed in PBS with 2% bovine calf serum and stained with streptavidin-PE-TexasRed secondary antibodies. DAPI was used for viability. Immunophenotypes were defined by signaling lymphocytic activation molecule (SLAM) family markers [35] CD48^−^CD150^+^LSK (SLAM LSK) as a population enriched for long-term HSCs. CD48^−^CD150^−^LSK and CD48^+^CD150^−^LSK populations are enriched for HPCs. Flow cytometry was performed on a LSR Fortessa analyzer (BD).

For cell sorting, bone marrow cells were first depleted of lineage-positive cells using biotinylated lineage cocktail and streptavidin-coupled Dynabeads (Invitrogen) per manufacturer's protocol. Lineage-negative (Lin^−^) cells were then stained for LSKs as described above and sorted on a FACS Aria Cell Sorter (BD). All analysis of FACS data was performed using FlowJo software (TreeStar).

### Colony-forming cell (CFC) assays

Unfractionated bone marrow cells or sorted LSK cells were plated at a concentration of 15,000 or 200 cells per plate, respectively, in duplicate using semisolid methylcellulose (Methocult M3434, StemCell Technologies) containing SCF, IL-3, IL-6 and erythropoietin. Cells were incubated at 37°C, 5% CO_2_ with high humidity, and colonies were enumerated after 10–12 days in culture.

### Competitive bone marrow transplantation (BMT), serial BMTs, and reverse BMTs

Bone marrow cells (CD45.2^+^) were harvested and sorted for LSK surface markers as described above. 500–1000 LSK cells were mixed with 350,000 competitor bone marrow cells (CD45.1^+^) and injected into each lethally-irradiated (a split dose of 10Gy, ^137^Cs source) [76] F1 recipient mouse (CD45.1^+^CD45.2^+^). Donor-derived reconstitution in the periphery was measured by flow cytometry every 4 weeks post-transplant. At 12–16 weeks, recipient mice were sacrificed and analyzed for donor HSPCs, as described above with the addition of CD45.1-PE-Cy7 and CD45.2-FITC antibodies. For secondary transplantation, 2×10^6^ unfractionated bone marrow cells from pooled primary recipient mice were transplanted into lethally irradiated F1 recipients. Reconstitution was again measured every 4 weeks post-transplant and data of secondary transplant endpoints are shown where mentioned. Tertiary transplants were performed similarly. In reverse BMTs, 1×10^6^ total bone marrow cells from CD45.1^+^ wild-type donor SJL mice were transplanted into lethally irradiated *f/f* and *f/−* control or *f/f;VEC* and *f/−;VEC* (CD45.2^+^) CKO recipients. Reconstitution and the percentage of CD45.1^+^ LSK and SLAM LSK cells were measured 8 weeks after transplantation.

## Supporting Information

S1 Fig **Deletion efficiency of Arap3^flox/flox^;Vav-Cre and Arap3^flox/flox^;VEC-Cre mice.** (A,B) Genotyping of individual colonies from CFC assays of *f/f;Vav* and *f/f;VEC* BMs. Representative PCR genotyping results from *f/f;Vav* (A) and *f/f;VEC* (B) colonies are shown. Controls are tail DNA isolated from control and CKO mice. The top band is floxed *Arap3* while the bottom band reflects deleted *Arap3*. (C) *Arap3* RNA transcript levels from *f/f;Vav* and *f/f;VEC* mice were assayed by qRT-PCR. The graph shows relative *Arap3* transcript levels remaining in the CKO mice compared to that in *f/f* controls. (D) Relative *Arap1* transcript levels (left) and *Arap2* transcript levels (right) to that in *f/f* mice. Each symbol represents an individual mouse; horizontal lines indicate mean ±SEM levels.
(TIF)

S2 Fig **Arap3^flox/flox^;Vav1-Cre mice maintain steady-state hematopoiesis and normal HSC function.** (A) CBC of *f/f* control (white bars) and *f/f;Vav* CKO mice (black bars). n = 17. (B) Flow cytometric analysis of the percentage of various cell populations in the BM, spleen, and thymus of *f/f* and *f/f;Vav* mice. n = 17. (C,D) CFC assays of *f/f* and *f/f;Vav* BM cells (C) and LSK cells (D) enumerated after 11 days in culture. n = 17 and 3, respectively. (E) Poly-RGD induced adherence of bone marrow-derived neutrophils from *f/f* and *f/f/;Vav* mice was quantified in triplicate as the number of cells per field of view at 20× magnification. (F–K) Serial transplantation of LSK cells from *f/f* and *f/f;Vav* donor mice. Results were pooled from three separate experiments. (F,I) Peripheral blood of recipient mice assessed at 4, 8, and 12 weeks after primary (F) or secondary (I) transplants for the percentage of donor-derived leukocytes. (G,J) Donor contribution to myeloid, B-cell, and T-cell compartments in the peripheral blood at the end of the primary (G) or secondary (J) transplants was analyzed by flow cytometry. (H,K) Bars show lineage distributions within donor-derived cells of individual recipient mice (left Y-axis), while diamond symbols indicate total donor leukocyte percentages (right Y-axis) in the peripheral blood, at the end of the primary (H) or secondary (K) transplants. T: CD3^+^; B: CD19^+^; M: Mac1^+^. Graphs show mean ±SEM. P-values determined by two-tailed Student's t-test.
(TIF)

S3 Fig **Arap3^flox/flox^;VEC-Cre mice show normal hematopoiesis and HSC functions.** (A) CBC of *f/f* control (white bars) and *f/f;VEC* CKO mice (gray bars). n = 12. (B) Percentage of various cell populations in the BM, spleen, and thymus of *f/f* and *f/f;VEC* mice. n = 12. (C) CFC assays of *f/f* and *f/f;VEC* BM cells enumerated after 11 days in culture. n = 12. (D–I) Serial transplantation of LSK cells from *f/f* and *f/f;VEC* donor mice. (D,G) Peripheral blood of recipient mice assessed every 4 weeks during the primary (D) or secondary (G) transplants for the percentage of donor-derived cells. (E,H) Donor contribution to myeloid, B-cell, and T-cell compartments in the peripheral blood after the primary (E) or secondary (H) transplants. (F,I) Bars show lineage distributions within donor-derived cells of individual recipient mice (left Y-axis), while diamond symbols indicate total donor leukocyte percentages (right Y-axis) in the peripheral blood, at the end of the primary (F) or secondary (I) transplants. Graphs show mean ±SEM. P-values determined by two-tailed Student's t-test.
(TIF)

## References

[1] Morrison SJ, Scadden DT (2014) The bone marrow niche for haematopoietic stem cells. Nature Rev 505:327–334. 10.1038/nature12984 PMC4514480 24429631

[2] Ding L, Morrison SJ (2013) Haematopoietic stem cells and early lymphoid progenitors occupy distinct bone marrow niches. Nature Lett 495:231–236. 10.1038/nature11885 PMC3600153 23434755

[3] Ding L, Saunders TL, Enikolopov G, Morrison SJ (2012) Endothelial and perivascular cells maintain haematopoietic stem cells. Nature 481:457–462. 22281595 10.1038/nature10783 PMC3270376

[4] Etienne-Manneville S, Hall A (2002) Rho GTPases in cell biology. Nature 420:629–635. 12478284 10.1038/nature01148

[5] Hall A (1998) Rho GTPases and the actin cytoskeleton. Science 279:509–514. 9438836 10.1126/science.279.5350.509

[6] Mulloy JC, Cancelas JA, Filippi MD, Kalfa TA, Guo F, et al (2010) Rho GTPases in hematopoiesis and hemopathies. Blood 115:936–947. 19965643 10.1182/blood-2009-09-198127 PMC2817638

[7] Gu Y, Filippi MD, Cancelas JA, Siefring JE, Williams EP, et al (2003) Hematopoietic cell regulation by Rac1 and Rac2 guanosine triphosphatases. Science 302:445–449. 14564009 10.1126/science.1088485

[8] Gottig S, Mobest D, Ruster B, Grace B, Winter S, et al (2006) Role of the monomeric GTPase Rho in hematopoietic progenitor cell migration and transplantation. Eur J Immunol 36:180–189. 16323242 10.1002/eji.200525607

[9] Ghiaur G, Lee AW, Bailey J, Cancelas JA, Zheng Y, et al (2006) Inhibition of RhoA GTPase activity enhances hematopoietic stem and progenitor cell proliferation and engraftment. Blood 108:2087–2094. 16709932 10.1182/blood-2006-02-001560

[10] Florian MC, Dorr K, Niebel A, Daria D, Schrenzenmeier H, et al (2012) Cdc42 activity regulates hematopoietic stem cell aging and rejuvenation. Cell Stem Cell 10:520–530. 22560076 10.1016/j.stem.2012.04.007 PMC3348626

[11] Chae H, Lee KE, Williams DA, Gu Y (2008) Cross-talk between RhoH and Rac1 in regulation of actin cytoskeleton and chemotaxis of hematopoietic progenitor cells. Blood 111:2597–2605. 18089848 10.1182/blood-2007-06-093237 PMC2254535

[12] Cancelas JA, Jansen M, Williams DA (2006) The role of chemokine activation of Rac GTPases in hematopoietic stem cell marrow homing, retention, and peripheral mobilization. Exp Hemat 34:976–985. 16863904 10.1016/j.exphem.2006.03.016

[13] Cancelas JA, Lee AW, Prabhakar R, Stringer KF, Zheng Y, et al (2005) Rac GTPases differentially integrate signals regulating hematopoietic stem cell localization. Nature Med 11:886–891. 16025125 10.1038/nm1274

[14] Zhang S, Zhou X, Lang RA, Guo F (2012) RhoA of the Rho family small GTPases is essential for B-cell lymphocyte development. PLoS One 7:e33773. 22438996 10.1371/journal.pone.0033773 PMC3306291

[15] Zhou X, Florian MC, Arumugam P, Chen X, Cancelas JA, et al (2013) RhoA GTPase controls cytokinesis and programmed necrosis of hematopoietic progenitors. J Exp Med 210:2371–2385. 24101377 10.1084/jem.20122348 PMC3804933

[16] Yang L, Wang L, Kalfa TA, Cancelas JA, Shang X, et al (2007) Cdc42 critically regulates the balance between myelopoiesis and erythropoiesis. Blood 110:3853–3861. 17702896 10.1182/blood-2007-03-079582 PMC2190607

[17] Guo F, Velu CS, Grimes HL, Zheng Y (2009) Rho GTPase Cdc42 is essential for B-lymphocyte development and activation. Blood 114:2909–2916. 19671922 10.1182/blood-2009-04-214676 PMC2756201

[18] Kalfa TA, Pushkaran S, Zhang X, Johnson JF, Pan D, et al (2010) Rac1 and Rac2 GTPases are necessary for early erythropoietic expansion in the bone marrow but not in the spleen. Hematologica 95:27–35. 10.3324/haematol.2009.006239 PMC2805739 20065081

[19] Wang L, Yang L, Filippi MD, Williams DA, Zheng Y (2006) Genetic deletion of Cdc42GAP reveals a role of Cdc42 in erythropoiesis and hematopoietic stem/progenitor cell survival, adhesion, and engraftment. Blood 107:98–105. 16174757 10.1182/blood-2005-05-2171 PMC1895350

[20] Xu H, Eleswarapu S, Geiger H, Szczur K, Daria D, et al (2009) Loss of the Rho GTPase activating protein p190-B enhances hematopoietic stem cell engraftment potential. Blood 114:3557–3566. 19713466 10.1182/blood-2009-02-205815 PMC2766675

[21] Raman R, Kumar RS, Hinge A, Kumar S, Nayak R, et al (2013) p190-B RhoGAP regulates the functional composition of the mesenchymal microenvironment. Leukemia 27:2209–2219. 23563238 10.1038/leu.2013.103 PMC3919554

[22] Krugmann S, Anderson KE, Ridley SH, Risso N, McGregor A, et al (2002) Identification of ARAP3, a novel PI3K effector regulating both Arf and Rho GTPases, by selective capture on phosphoinositide affinity matrices. Mol Cell 9:95–108. 11804589 10.1016/s1097-2765(02)00434-3

[23] Krugmann S, Williams R, Stephens LR, Hawkins PT (2004) ARAP3 is a PI3K- and Rap-regulated GAP for RhoA. Curr Biol 14:1380–1384. 15296756 10.1016/j.cub.2004.07.058

[24] Krugmann S, Stephens LR, Hawkins PT (2006) Purification of ARAP3 and characterization of GAP activities. Methods Enzymol 406:91–103. 16472652 10.1016/S0076-6879(06)06008-3

[25] I STT, Nie Z, Stewart A, Najdovska M, Hall NE, et al (2004) ARAP3 is transiently tyrosine phosphorylated in cells attaching to fibronectin and inhibits cell spreading in a RhoGAP-dependent manner. J Cell Sci 117:6071–6084. 15546919 10.1242/jcs.01526

[26] Krugmann S, Andrews S, Stephens L, Hawkins PT (2005) ARAP3 is essential for formation of lamellipodia after growth factor stimulation. J Cell Sci 119:425–432. 10.1242/jcs.02755 16418224

[27] Xu J, Wang F, Van Keymeulen A, Herzmark P, Straight A, et al (2003) Divergent signals and cytoskeletal assemblies regulate self-organizing polarity in neutrophils. Cell 114:201–214. 12887922 10.1016/s0092-8674(03)00555-5

[28] Del Pozo MA, Vicente-Manzanares M, Tejedor R, Serrador JM, Sanchez-Madrid F (1999) Rho GTPases control migration and polarization of adhesion molecules and cytoskeletal ERM components in T lymphocytes. Eur J Immunol 29:3609–3620. 10556816 10.1002/(SICI)1521-4141(199911)29:11<3609::AID-IMMU3609>3.0.CO;2-S

[29] Gambardella L, Hemberger M, Hughes B, Zudaire E, Andrews S, et al (2010) PI3K signaling through the dual GTPase-activating protein ARAP3 is essential for developmental angiogenesis. Sci STKE 3:ra76. 10.1126/scisignal.2001026 20978237

[30] Kartopawiro J, Bower NI, Karnezis T, Kazenwadel J, Betterman KL, et al (2014) Arap3 is dysregulated in a mouse model of hypotrichosis-lymphedema-telangiectasia and regulates lymphatic vascular development. Hum Mol Genet 23:1286–1297. 24163130 10.1093/hmg/ddt518

[31] Dzierzak E, Speck NA (2008) Of lineage and legacy: the development of mammalian hematopoietic stem cells. Nature Immunol 9:129–136. 18204427 10.1038/ni1560 PMC2696344

[32] Gambardella L, Anderson KE, Nussbaum C, Segonds-Pichon A, Margarido T, et al (2011) The GTPase-activating protein ARAP3 regulates chemotaxis and adhesion-dependent processes in neutrophils. Blood 118:1087–1098. 21490342 10.1182/blood-2010-10-312959

[33] Gambardella L, Anderson KE, Jakus Z, Kovacs M, Voigt S, et al (2013) Phosphoinositide 3-OH Kinase regulates integrin-dependent processes in neutrophils by signaling through its effector ARAP3. J Immunol 190:381–391. 23180820 10.4049/jimmunol.1201330 PMC3672969

[34] Craig HE, Coadwell J, Guillou H, Vermeren S (2010) ARAP3 binding to phosphatidylinositol-(3,4,5)-triphosphate depends on N-terminal tandem PH domains and adjacent sequences. Cell Signal 22:257–264. 19786092 10.1016/j.cellsig.2009.09.025

[35] Kiel MJ, Yilmaz OH, Iwashita T, Yilmaz OH, Terhorst C, et al (2005) SLAM family receptors distinguish hematopoietic stem and progenitor cells and reveal endothelial niches for stem cells. Cell 121:1109–1121. 15989959 10.1016/j.cell.2005.05.026

[36] Janzen V, Forkert R, Fleming HE, Saito Y, Waring MT, et al (2006) Stem-cell ageing modified by the cyclin-dependent kinase inhibitor p16INK4a. Nature 443:421–426. 16957735 10.1038/nature05159

[37] Rossi DJ, Bryder D, Zahn JM, Ahlenius H, Sonu R, et al (2005) Cell intrinsic alterations underlie hematopoietic stem cell aging. Proc Natl Acad Sci 102:9194–9199. 15967997 10.1073/pnas.0503280102 PMC1153718

[38] Bersenev A, Rozenova K, Balcerek J, Jiang J, Wu C, et al (2012) Lnk deficiency partially mitigates hematopoietic stem cell aging. Aging Cell 11:949–959. 22812478 10.1111/j.1474-9726.2012.00862.x PMC3500428

[39] Stadtfeld M, Graf T (2004) Assessing the role of hematopoietic plasticity for endothelial and hepatocyte development by non-invasive lineage tracing. Dev Biol 132:203–213. 10.1242/dev.01558 15576407

[40] Greenbaum A, Hsu YS, Day RB, Schuettpelz LG, Christopher MJ, et al (2013) CXCL12 in early mesenchymal progenitors is required for haematopoietic stem-cell maintenance. Nature Lett 495:227–231. 10.1038/nature11926 PMC3600148 23434756

[41] Kisanuki YY, Hammer RE, Miyazaki J, Williams SC, Richardson RA, et al (2001) Tie2-Cre transgenic mice: a new model for endothelial cell-lineage analysis in vivo. Dev Biol 230:230–242. 11161575 10.1006/dbio.2000.0106

[42] Iurlaro M, Scatena M, Zhu W, Fogel E, Wieting SL, et al (2003) Rat aorta-derived mural precursor cells express the Tie2 receptor and respond directly to stimulation by angiopoietins. J Cell Sci 116:3635–3643. 12876214 10.1242/jcs.00629

[43] Alva JA, Zovein AC, Monvoisin A, Murphy T, Salazar A, et al (2006) VE-Cadherin-Cre-Recombinase transgenic mouse: a tool for lineage analysis and gene deletion in endothelial cells. Dev Dyna 235:759–767. 10.1002/dvdy.20643 16450386

[44] Chen MJ, Yokomizo T, Zeigler BM, Dzierzak E, Speck NA (2009) Runx1 is required for the endothelial to haematopoietic cell transition but not thereafter. Nature 457:887–892. 19129762 10.1038/nature07619 PMC2744041

[45] Leko V, Varnum-Finney B, Li H, Gu Y, Flowers D, et al (2012) SIRT1 is dispensable for function of hematopoietic stem cells in adult mice. Blood 119:1856–1860. 22219225 10.1182/blood-2011-09-377077 PMC3293640

[46] Singh SK, Williams CA, Klarmann K, Burkett SS, Keller JR, et al (2013) Sirt1 ablation promotes stress-induced loss of epigenetic and genomic hematopoietic stem and progenitor cell maintenance. J Exp Med 210:987–1001. 23630229 10.1084/jem.20121608 PMC3646499

[47] Kuhn R, Schwenk F, Aguet M, Rajewsky K (1995) Inducible gene targeting in mice. Science 269:1427–1429. 7660125 10.1126/science.7660125

[48] Miura K, Jacques KM, Stauffer S, Kubosaki A, Zhu K, et al (2002) ARAP1: A point of convergence for Arf and Rho signaling. Mol Cell 9:109–119. 11804590 10.1016/s1097-2765(02)00428-8

[49] Cuthbert EJ, Davis KK, Casanova JE (2007) Substrate specificities and activities of AZAP family Arf GAPs in vivo. Am J Physiol Cell Physiol 294:C263–C270. 18003747 10.1152/ajpcell.00292.2007

[50] Yoon HY, Miura K, Cuthbert EJ, Davis KK, Ahvazi B, et al (2006) ARAP2 effects on the actin cytoskeleton are dependent on Arf6-specific GTPase-activating-protein activity and binding to RhoA-GTP. J Cell Sci 119:4650–4666. 17077126 10.1242/jcs.03237

[51] Kowanetz K, Husnjak K, Holler D, Kowanetz M, Soubeyran P, et al (2004) CIN85 associates with multiple effectors controlling intracellular trafficking of epidermal growth factor receptors. Mol Biol Cell 15:3155–3166. 15090612 10.1091/mbc.E03-09-0683 PMC452573

[52] Wu B, Wang F, Zhang J, Zhang Z, Qin L, et al (2012) Identification and structural basis for a novel interaction between Vav2 and Arap3. J Struct Biol 180:84–95. 22750419 10.1016/j.jsb.2012.06.011

[53] Leone M, Cellitti J, Pellecchia M (2009) The Sam domain of the lipid phosphatase Ship2 adopts a common model to interact with Arap3-Sam and EphA2-Sam. BMC Struct Biol 9:59. 19765305 10.1186/1472-6807-9-59 PMC2755476

[54] Raaijmakers JH, Deneubourg L, Rehmann H, de Koning J, Zhang Z, et al (2007) The PI3K effector Arap3 interacts with the PI(3,4,5)P3 phosphatase SHIP2 in a SAM domain-dependent manner. Cell Signal 19:1249–1257. 17314030 10.1016/j.cellsig.2006.12.015

[55] Mercurio FA, Marasco D, Pirone L, Scognamiglio PL, Pedone EM, et al (2013) Heterotypic Sam-Sam association between Odin-Sam1 and Arap3-Sam: binding affinity and structural insights. Chembiochem 14:100–106. 23239578 10.1002/cbic.201200592 PMC3631714

[56] Pesesse X, Moreau C, Drayer AL, Woscholski R, Parker P, et al (1998) The SH2 domain containing inositol 5-phosphatase SHIP2 displays phosphatidylinositol 3,4,5-trisphosphate and inositol 1,3,4,5-tetrakisphosphate 5-phosphatase activity. FEBS Lett 437:301–303. 9824312 10.1016/s0014-5793(98)01255-1

[57] Zheng X, Zhang J, Liao K (2014) The basic amino acids in the coiled-coil domain of CIN85 regulate its interaction with c-Cbl and phosphatidic acid during epidermal growth factor receptor (EGFR) endocytosis. BMC Biochem 15:13. 25005938 10.1186/1471-2091-15-13 PMC4096430

[58] Tossidou I, Niedenthal R, Klaus M, Teng B, Worthmann K, et al (2012) CD2AP regulates SUMOylation of CIN85 in podocytes. Mol Cell Biol 32:1068–1079. 22203040 10.1128/MCB.06106-11 PMC3295011

[59] Tong J, Sydorskyy Y, St-Germain JR, Taylor P, Tsao MS, et al (2013) Odin (ANKS1A) modulates EGF receptor recycling and stability. PLoS One 8:e64817. 23825523 10.1371/journal.pone.0064817 PMC3692516

[60] Jeon CY, Kim HJ, Morii H, Mori N, Settleman J, et al (2010) Neurite outgrowth from PC12 cells by basic fibroblast growth factor (bFGF) is mediated by RhoA inactivation through p190RhoGAP and ARAP3. J Cell Physiol 224:786–794. 20578246 10.1002/jcp.22184

[61] Randazzo PA, Hirsch DS (2004) Arf GAPs: multifunctional proteins that regulate membrane traffic and actin remodeling. Cell Signal 16:401–413. 14709330 10.1016/j.cellsig.2003.09.012

[62] Nie Z, Randazzo PA (2006) Arf GAPs and membrane traffic. J Cell Sci 119:1203–1211. 16554436 10.1242/jcs.02924

[63] Randazzo PA, Inoue H, Bharti S (2007) Arf GAPs as regulators of the actin cytoskeleton. Biol Cell 99:583–600. 17868031 10.1042/bc20070034

[64] Schweitzer JK, D'Souza-Schorey C (2005) A requirement for Arf6 during the completion of cytokinesis. Exp Cell Res 311:74–83. 16181626 10.1016/j.yexcr.2005.07.033

[65] Knizhnik AV, Kovaleva OV, Komelkov AV, Trukhanova LS, Rybko VA, et al (2011) Arf6 promotes cell proliferation via the PLD-mTORC1 and p38MAPK pathways. J Cell Biochem 113:360–371. 10.1002/jcb.23362 21928324

[66] Randazzo PA, Nie Z, Miura K, Hsu VW (2000) Molecular aspects of the cellular activities of ADP-ribosylation factors. Sci STKE 2000:re1. 10.1126/stke.2000.59.re1 11752622

[67] Choi W, Karim ZA, Whiteheart SW (2006) Arf6 plays an early role in platelet activation by collagen and convulxin. Blood 107:3145–3152. 16352809 10.1182/blood-2005-09-3563 PMC1895749

[68] Lu Q, Wei W, Kowalski PE, Chang AY, Cohen SN (2004) EST-based genome-wide gene inactivation identifies ARAP3 as a host protein affecting cellular susceptibility to anthrax toxin. PNAS 101:17246–17251. 15569923 10.1073/pnas.0407794101 PMC534609

[69] Nandy D, Asmann YW, Mukhopadhyay D, Basu A (2009) Role of AKT-glycogen synthase kinase axis in monocyte activation in human beings with and without type 2 diabetes. J Cell Mol Med 14:1396–1407. 19754670 10.1111/j.1582-4934.2009.00900.x PMC2912967

[70] Yagi R, Tanaka M, Sasaki K, Kamata R, Nakanishi Y, et al (2011) ARAP3 inhibits peritoneal dissemination of scirrhous gastric carcinoma cells by regulating cell adhesion and invasion. Oncogene 30:1413–1421. 21076469 10.1038/onc.2010.522

[71] Cherfils J, Zeghouf M (2013) Regulation of small GTPases by GEFs, GAPs, and GDIs. Physiol Rev 93:269–309. 23303910 10.1152/physrev.00003.2012

[72] Karlsson R, Pedersen ED, Wang Z, Brakebusch C (2009) Rho GTPase function in tumorigenesis. Biochim Biophys Acta 1796:91–98. 19327386 10.1016/j.bbcan.2009.03.003

[73] Hall A (2009) The cytoskeleton and cancer. Cancer Metastasis Rev 28:5–14. 19153674 10.1007/s10555-008-9166-3

[74] Lazer G, Katzav S (2011) Guanine nucleotide exchange factors for RhoGTPases: good therapeutic targets for cancer therapy? Cell Signal 23:969–979. 21044680 10.1016/j.cellsig.2010.10.022

[75] Troeger A, Williams DA (2013) Hematopoietic-specific Rho GTPases Rac2 and RhoH and human blood disorders. Exp Cell Res 319:2375–2383. 23850828 10.1016/j.yexcr.2013.07.002 PMC3997055

[76] Bersenev A, Wu C, Balcerek J, Tong W (2008) Lnk controls mouse hematopoietic stem cell self-renewal and quiescence through direct interactions with JAK2. J Clin Invest 118:2832–2844. 18618018 10.1172/JCI35808 PMC2447929

