# Supplementary figures and images for: ARAP3 Functions in Hematopoietic Stem Cells
Source: PLoS One. 2014 Dec 26;9(12):e116107. doi: 10.1371/journal.pone.0116107 (PMC4277471; doi:10.1371/journal.pone.0116107)

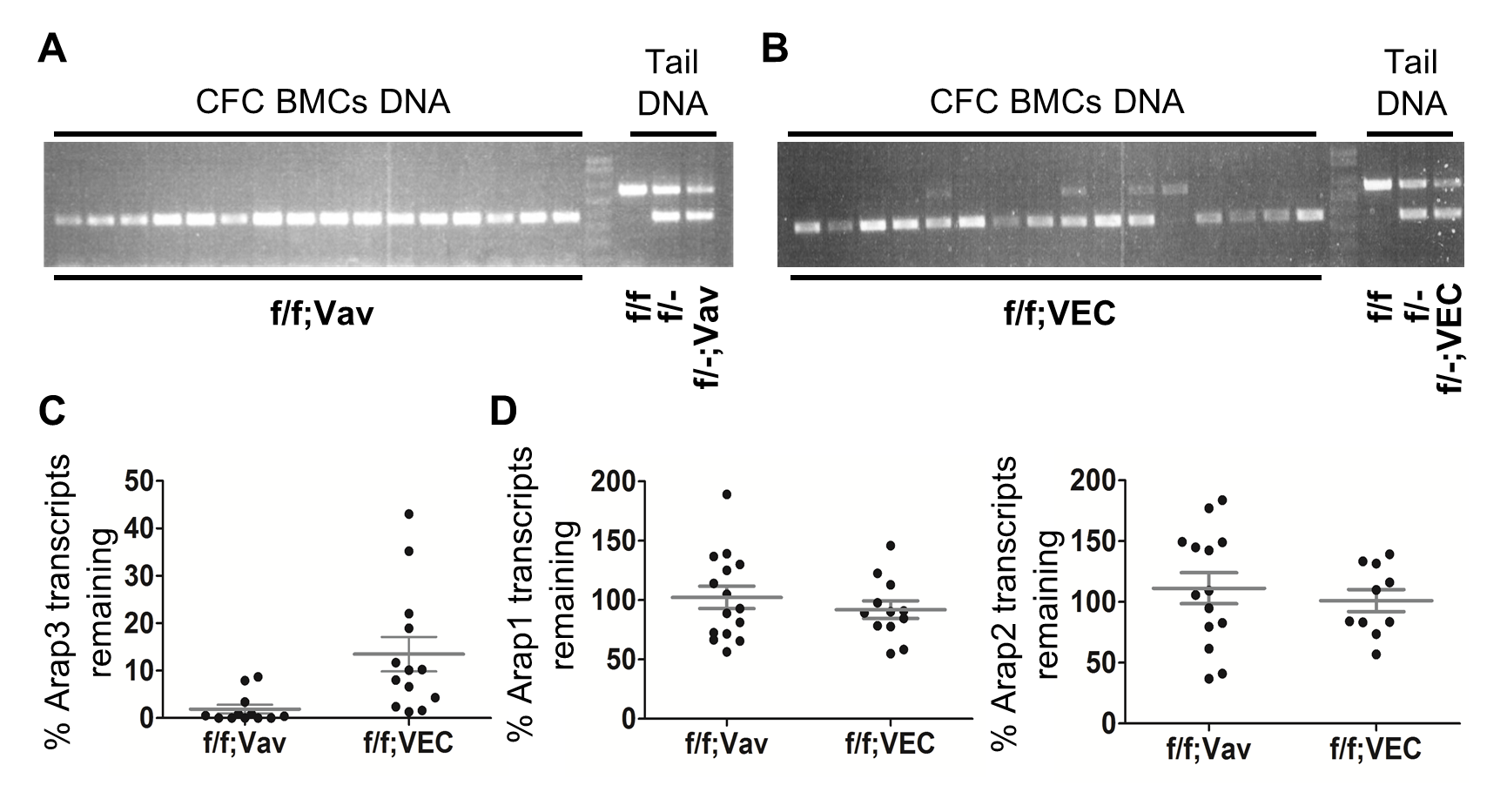

Supplement: S1 Fig — Deletion efficiency of Arap3flox/flox;Vav-Cre and Arap3flox/flox;VEC-Cre mice. (A,B) Genotyping of individual colonies from CFC assays of f/f;Vav and f/f;VEC BMs. Representative PCR genotyping results from f/f;Vav (A) and f/f;VEC (B) colonies are shown. Controls are tail DNA isolated from control and CKO mice. The top band is floxed Arap3 while the bottom band reflects deleted Arap3. (C) Arap3 RNA transcript levels from f/f;Vav and f/f;VEC mice were assayed by qRT-PCR. The graph shows relative Arap3 transcript levels remaining in the CKO mice compared to that in f/f controls. (D) Relative Arap1 transcript levels (left) and Arap2 transcript levels (right) to that in f/f mice. Each symbol represents an individual mouse; horizontal lines indicate mean ±SEM levels. (TIF) [file pone.0116107.s001.tif]
